# Structural, Morphological, and Textural Characterization of Ni- and Fe-Based KCC-1@C Hybrid Nanocomposites

**DOI:** 10.3390/nano16171068

**Published:** 2026-08-27

**Authors:** Luz D. Balbin-Córdoba, Julián Vanegas-Ramirez, Lorena Marín, Luis A. Rodríguez, César Magén, Jesús A. Tabares, Milton Manotas-Albor, Renso Visbal, Malka Mora

**Affiliations:** 1 Departamento de Química, Facultad de Ciencias Naturales y Exactas, Universidad del Valle, Cali 760032, Colombia; luz.balbin@correounivalle.edu.co (L.D.B.-C.); julian.vanegas@correounivalle.edu.co (J.V.-R.); 2 Centro de Excelencia en Nuevos Materiales (CENM), Universidad del Valle, Cali 760032, Colombia; marin.lorena@correounivalle.edu.co (L.M.); luis.a.rodriguez@correounivalle.edu.co (L.A.R.); jesus.tabares@correounivalle.edu.co (J.A.T.); 3 Grupo de Películas Delgadas (GPD), Departamento de Física, Universidad del Valle, Cali 760032, Colombia; 4 Dirección de Laboratorios, Servicio Geológico Colombiano, Cali 760031, Colombia; 5 Grupo de Transiciones de Fase y Materiales Funcionales, Departamento de Física, Facultad de Ciencias Naturales y Exactas, Universidad del Valle, Cali 760032, Colombia; 6 Instituto de Nanociencia y Materiales de Aragón (INMA), CSIC-Universidad de Zaragoza, 50009 Zaragoza, Spain; cmagend@unizar.es; 7 Grupo de Metalúrgica Física y Teoría de Transiciones de Fase (GMTF), Departamento de Física, Universidad del Valle, Cali 760032, Colombia; 8 Grupo de Investigación en Física Aplicada (GIFA), Departamento de Física y Geociencias, Universidad del Norte, Barranquilla 080001, Colombia; manotasm@uninorte.edu.co

**Keywords:** polymer-assisted deposition (PAD), metal dispersion, resin, catalysis, DFNS

## Abstract

Hybrid nanocomposites composed of dendritic fibrous nanosilica (DFNS) identified as KCC-1 and resorcinol–formaldehyde (RF) resin were synthesized via the polymer-assisted deposition (PAD) method for the incorporation of Ni and Fe in mono- and bimetallic configurations. The RF resin enabled uniform dispersion Fe-based and Ni-based nanoparticles through coordination with functional groups, while KCC-1 provided a high-surface-area, mesoporous support. A comprehensive structural and chemical characterization confirmed the formation of hematite in the Fe-based system; NiO and metallic Ni in the Ni-based material; and FeO, NiO and metallic Ni in the bimetallic composite, in the form of well-dispersed metal nanoparticles between 3–6 nm. Although a reduction in BET surface area was observed due to resin and metal loading, all nanocomposites retained mesoporous structures with Type IV isotherms and H3-type hysteresis, suitable for catalytic applications. The nanocomposites exhibited structural stability up to 800 °C under N_2_, with a total mass loss of 12–14% dominated by moisture desorption below 100 °C (~8–11%) and minor surface group degradation at higher temperatures. These findings demonstrate the potential of the PAD method for fabricating functional hybrid materials with improved metal dispersion.

## 1. Introduction

In recent years, the development of hybrid or composite nanomaterials has gained significant attention due to their enhanced physicochemical properties and multifunctional performance. These composites typically combine two or more distinct nanostructured materials, such as metals, metal oxides, polymers, or porous supports, which interact synergistically to overcome the limitations of their individual constituents. The rational design of hybrid nanomaterials enables the tuning of structural, electronic, and surface properties, thereby improving their functionality in diverse fields, such as water and wastewater treatment, catalysis and electronic applications [1,2].

One of the key advantages of hybrid nanomaterials lies in their ability to integrate complementary properties from different domains. For instance, inorganic components such as mesoporous silicas or metal oxides offer high thermal stability, mechanical robustness, and well-defined porous structures, while organic phases like polymers or carbonaceous materials provide chemical versatility, hydrophobicity, and tunable surface functionalities. When combined, these features can significantly improve charge transfer, mass transport, and surface reactivity, attributes that are particularly desirable in catalytic, environmental, sensor, biomedical and energy-related applications [3,4,5,6,7].

Mesoporous silica materials have attracted substantial attention in material science due to their versatile structural architectures, high surface areas, and tunable pore dynamics. Traditional mesoporous silicas, such as MCM-41 and SBA-15, typically possess ordered, cylindrical pore frameworks; however, their restricted pore accessibility often leads to severe mass-transfer limitations and site blockage during chemical transformations or active species loading. To address these drawbacks, Polshettiwar and co-workers engineered dendritic fibrous nanosilica (DFNS), also widely designated as KCC-1 [8]. Unlike conventional porous silicas, DFNS features an open, center-radial dendritic architecture composed of three-dimensionally oriented silica fibers or sheets that radiate outward from the particle core. This unique morphology ensures complete accessibility to the internal surface area from all spatial angles, significantly mitigating diffusion resistance. The structural parameters of DFNS can be precisely modulated during synthesis, enabling tailored performance across diverse operational regimes. DFNS exhibits variable particle diameters ranging from 50 to 1200 nm, exceptional specific surface areas spanning 500 to 1244 m^2^·g^−1^, high pore volumes up to 2.18 cm^3^·g^−1^, and broad pore size distributions (3.7–25 nm) formed by the inter-fiber spaces. Furthermore, the dense, interconnecting network of covalently bonded silica sheets imparts remarkable hydrothermal and thermal stability up to 800 °C, alongside high mechanical resilience under pressures reaching 130 MPa [8,9].

Owing to its accessible open-pore structure and facile surface functionalization, DFNS has emerged as a robust platform for critical energy and environmental technologies. Prominent applications include heterogeneous catalysis and photocatalysis, where DFNS serves as an efficient support for single-atom catalysts, plasmonic metal nanoparticles, and solid acid/base sites. In environmental remediation, functionalized DFNS derivatives demonstrate superior performance in direct CO_2_ capture, high-temperature sorbent engineering, and the catalytic reduction of CO_2_ into valuable solar fuels or methane. Furthermore, its high functional group loading capacity makes DFNS an effective nanoadsorbent for hazardous water pollutants, including heavy metals and organic dyes, among other applications [8,9,10].

Although direct deposition of metal nanoparticles onto mesoporous SiO_2_ has been extensively reported, these methods generally rely on the limited density of surface silanol groups to anchor metal precursors, which may lead to non-uniform nucleation, broader particle-size distributions, and metal sintering during subsequent thermal treatments, particularly at moderate or high metal loadings. In contrast, the incorporation of resorcinol–formaldehyde (RF) resin provides an active polymeric coordination matrix that fundamentally modifies the nucleation and growth mechanism of the metallic phase rather than simply acting as a sacrificial intermediate. During the base-catalyzed polycondensation of resorcinol and formaldehyde, a highly cross-linked three-dimensional polymeric network is generated, containing abundant phenolic hydroxyl groups and π-conjugated aromatic rings that exhibit strong coordination ability toward transition-metal ions through metal–oxygen interactions, hydrogen bonding, and π-electron donation. As demonstrated by Wang et al., the RF coating uniformly covers the silica surface and creates a homogeneous distribution of oxygen-containing functional groups, substantially increasing the number of anchoring sites available for metal precursors compared with bare silica [11]. This homogeneous chemical environment promotes the formation of spatially separated nucleation centers, suppresses local precursor concentration gradients, and consequently inhibits nanoparticle coalescence during reduction. Likewise, Chen and Shen demonstrated that incorporating an RF resin during the preparation of Co/SiO_2_ catalysts significantly enhanced metal dispersion and reducibility relative to conventional impregnation, resulting in smaller metallic particles and a higher density of accessible active sites [12]. Furthermore, the conformal polymer layer surrounding the silica framework generates a confinement effect that restricts the diffusion and migration of metal species during calcination and reduction, thereby minimizing thermally induced sintering. After pyrolysis, the RF resin is transformed into a porous carbonaceous matrix that largely preserves the initial spatial distribution of the metal species while providing additional structural stability and stronger metal–support interactions. Zhang et al. further demonstrated that RF-derived matrices efficiently stabilize noble-metal nanoparticles, preventing aggregation and enabling the formation of highly dispersed metal nanostructures with narrow particle-size distributions [13]. Therefore, the purpose of incorporating RF resin in the present work is not simply to facilitate the deposition of metal nanoparticles onto SiO_2_, which is indeed achievable by conventional methods, but rather to regulate precursor coordination, nucleation kinetics, nanoparticle growth, and thermal stability at the molecular level for potential applications in catalysis and water treatment, among others. Despite the great number of works reporting on the structural characterization and applications of KCC-1 [8,9] and RF resin [14,15] for the construction of functional materials, to the best of our knowledge, there are no works reporting on the preparation of KCC-1-RF resin hybrid materials. Only recent studies by Ali et al. (2017) [16] report the synthesis of mesoporous yolk–shell nanoparticles (MYSNs) using a template-assisted etching method in which RF resin served as a sacrificial layer. Initially, Fe_3_O_4_ nanoparticles were coated with SiO_2_ and subsequently enveloped with an RF shell, forming Fe_3_O_4_@SiO_2_@RF composites. A mesoporous silica layer was then grown on the outer surface, and selective removal of RF and CTAB yielded two yolk–shell structures: one with a fibrous KCC-1-like morphology and another with conventional mesoporous silica. In this case, the use of RF enabled the formation of hollow interiors and large mesopores (20–50 nm), providing high surface areas (up to 625 m^2^·g^−1^) and facilitating effective enzyme immobilization [16]. Thus, by combining these two components, KCC-1 and RF resin, a hybrid support material can be engineered with synergistic advantages. The use of RF resin as a polymeric medium for metal incorporation onto KCC-1 offers a promising strategy to improve metal dispersion. The functional groups within the RF matrix can coordinate metal ions, facilitating their uniform distribution throughout the support [17]. This approach can help to minimize the formation of metal aggregates during synthesis and thermal treatment, potentially enhancing the accessibility of active sites and the overall performance of the resulting hybrid nanocomposite.

The concept of polymer-assisted deposition (PAD) has emerged as a powerful strategy to immobilize metal precursors onto support surfaces while improving their dispersion and controlling particle size at the nanoscale. Traditionally, PAD involves the use of soluble polymers such as polyethyleneimine (PEI), polyacrylic acid (PAA), or other water-soluble organic molecules, which act as scaffolds or stabilizing agents for metal ions using a “pre-complexation, then deposition” sequence. In conventional approaches, solid supports (e.g., KCC-1) are directly impregnated with metal salts, which frequently leads to localized metal aggregation and poor dispersion during drying and activation. In contrast, the PAD strategy ensures that metal ions are first complexed with the polymer precursor in solution to form stable, homogeneous metal–polymer species [18]. Upon calcination or reduction, the metal–polymer complexes decompose, leading to the formation of finely dispersed nanoparticles with narrow size distribution. This sequential control prevents premature metal sintering and ensures a significantly more uniform metal distribution across the material. This technique has shown great promise in enhancing the catalytic activity, selectivity, and durability of supported metal systems in various reactions, particularly in hydrogenation, oxidation, and electrochemical applications [18].

In this work, we propose a modulation of the PAD strategy by employing RF resin as a polymeric host for Ni, Fe and Ni-Fe incorporation, instead of conventional polymers. The phenolic structure of RF resin can allow for strong chelation with transition metal ions, promoting intimate contact and uniform distribution prior to thermal processing. Moreover, the carbonization of RF under inert conditions can lead to a conductive, porous matrix that further stabilizes metal nanoparticles and prevents sintering. By integrating RF-functionalized metal species onto the dendritic structure of KCC-1, we aim to produce nanocomposites with a moderate surface area, hierarchical porosity, and superior metal dispersion, but with a high thermal stability provided by the presence of the KCC-1 template.

## 2. Materials and Methods

### 2.1. Chemicals

The surfactant, cetylpyridinium bromide (CPB, >99%), silica precursor, tetraethylorthosilicate (TEOS, >98%), 1-pentanol, resorcinol, 37% aqueous formaldehyde solution, ethanol, sodium carbonate, nickel precursor [Ni(OAc)_2_∙4H_2_O] and iron precursor [Fe(NO_3_)_3_∙9H_2_O] were purchased from Sigma Aldrich, Saint Louis, MO, USA. Cyclohexane and urea were supplied by PanReac, Barcelona, Spain. All of the chemicals were used as received without further purification. Deionized water was used in all experiments.

### 2.2. Preparation of Dendritic Fibrous Silica Nanospheres (KCC-1)

KCC-1 was adapted and prepared according to a previous work [19]. Briefly, TEOS (5.3 mL, 0.024 mmol) was dispersed into a round-bottom flask containing cyclohexane (70 mL) and 1-pentanol (5.3 mL), referred to as solution A. Similarly, CPB (2 g, 0.052 mmol) and urea (2.6 g, 0.043 mmol) were dissolved in 30 mL of deionized water, referred to as solution B. Solution A was homogeneously mixed in B, stirred at room temperature for 30 min and refluxed for 4 h. The solution obtained was dried in an oven overnight. Finally, the prepared samples were heat treated in a muffle furnace at 550 °C for 6 h to obtain KCC-1 [20,21].

### 2.3. Preparation of Resorcinol-Formaldehyde (RF) Resin

A total of 0.625 g of resorcinol was dissolved in 4 mL of deionized water with 4 mL of ethanol. To this solution, 1.6 mL of 37% aqueous formaldehyde solution was added under constant stirring. Finally, 0.03 g of sodium carbonate catalyst was added to the solution under constant stirring at 50 °C temperature. Stirring continued for approximately 40 min. Finally, to avoid solidification of the resin, 25 mL of ethanol was added [22,23].

### 2.4. Impregnation of the Resin in the KCC-1 Support

To 0.500 g of KCC-1 support, 2 mL of the prepared dissolved RF resin was added dropwise and then dried in an oven overnight. Subsequently, it was calcined in a tube furnace in N_2_ atmosphere with a ramp of 5 °C/min at 600 °C for 3 h.

### 2.5. Preparation of Ni/KCC-1@C, Fe/KCC-1@C and Ni-Fe/KCC-1@C Nanocomposites by PAD

The hybrid nanocomposites were synthesized through a polymer-assisted deposition method. In a typical synthesis, the corresponding metal precursors were dissolved in 5 mL of distilled water under continuous stirring for 10 min. Subsequently, 2.0 mL of an RF resin solution (previously diluted in ethanol) was added to the metal solution, and the mixture was stirred for an additional 20 min at 40 °C.

The silica-based support KCC-1 (0.500 g) was then incorporated into the solution, and the mixture was maintained under constant stirring at 60 °C until a dry solid was obtained. The resulting material was dried overnight in an oven at 60 °C to ensure complete solvent removal. Finally, the dry solids were subjected to a thermal treatment in a tubular furnace under nitrogen atmosphere at 600 °C for 3 h, using a heating ramp of 5 °C·min^−1^ [24,25].

Three different metal loadings were prepared:

Monometallic 5 wt% Ni/KCC-1@C: 0.1110 g of ([Ni(OAc)_2_·4H_2_O], 0.446 mmol);
Monometallic 5 wt% Fe/KCC-1@C: 0.1903 g of ([Fe(NO_3_)_3_·9H_2_O], 0.471 mmol);
Bimetallic 5 wt% Ni–5 wt% Fe/KCC-1@C: 0.1060 g of [Ni(OAc)_2_·4H_2_O] (0.426 mmol) and 0.1810 g of [Fe(NO_3_)_3_·9H_2_O] (0.448 mmol).

In the case of the bimetallic system, the Fe and Ni precursors were co-dissolved prior to the addition of the resin, ensuring their homogeneous distribution within the organic matrix before deposition onto the silica support. This co-impregnation approach promotes intimate contact between the metal species and the RF matrix, which may influence the final porosity and dispersion upon carbonization.

### 2.6. Characterization of Nanocomposites

Fourier transform infrared spectroscopy (FTIR) of nanocomposites was carried out on a JASCO FT-IR spectrophotometer, Easton, MD, USA. Raman Spectroscopy was performed on an NRS-4500 JASCO, with the laser wavelength set at 784.98 nm and a laser power of 6.6 mW. Each spectrum was obtained by averaging two exposures over 30 s. Wide-angle X-ray diffraction (XRD) spectra were recorded using a Bruker D8 Advance Eco diffractometer, Karlsruhe, Germany equipped with a Cu anode (λ = 0.1540 nm) in a 2θ range of 10–80° (0.01° scan rate). The operating voltage and current were set at 40 kV and 25 mA, respectively. The assignment of diffraction peaks was achieved using the X’pert HighScore Plus software (v3.0). Mössbauer transmission spectra were measured at room temperature using a constant acceleration spectrometer with a ^57^Co/Rh radioactive source of ~5 mCi. Mössbauer spectra were fitted using the MOSFIT software (v2016 1.0) [26], and the isomer shift (IS) was measured with respect to the α-Fe. Transmission electron microscopy (TEM) was used to analyze the crystal structure, morphology, and elemental composition of the nanocomposites at the atomic and nanoscale levels. TEM specimens were prepared by depositing a drop of hybrid composites powder dispersed in ethanol on Cu-supported holey carbon grids. High-resolution transmission electron microscopy (HRTEM) images were acquired using an image-corrected FEI Titan^3^ microscope (Waltham, MA, USA). Scanning transmission electron microscopy (STEM) in high-angle annular dark field (HAADF) mode and energy-dispersive X-ray spectroscopy (EDS) analyses were conducted using a probe-corrected FEI Titan Low Base microscope, equipped with an Ultim Max TLE10 EDS spectrometer by Oxford Instruments, High Wycombe, United Kingdom. Both instruments were operated at 300 kV. Fast Fourier transform (FFT) analysis was applied to a selected HRTEM image using the Gatan Digital Micrograph software. The Brunauer–Emmett–Teller (BET) surface area of the hybrid nanocomposites was calculated by the N_2_ sorption method; meanwhile, the pore size and pore volume were obtained by the Barrette–Joynere–Halenda (BJH) model on a Quantachrome NOVA 1000e (Boynton Beach, FL, USA). Before this analysis, the samples were outgassed at 300 °C for 4 h. Thermogravimetric analysis (TGA) was performed in the same SDT-650 TA Instruments setup, at a heating rate of 5 °C/min, in a nitrogen flow rate of 100 mL/min, up to a maximum temperature of 800 °C.

## 3. Results and Discussion

### 3.1. Synthesis Overview

**Scheme 1:**
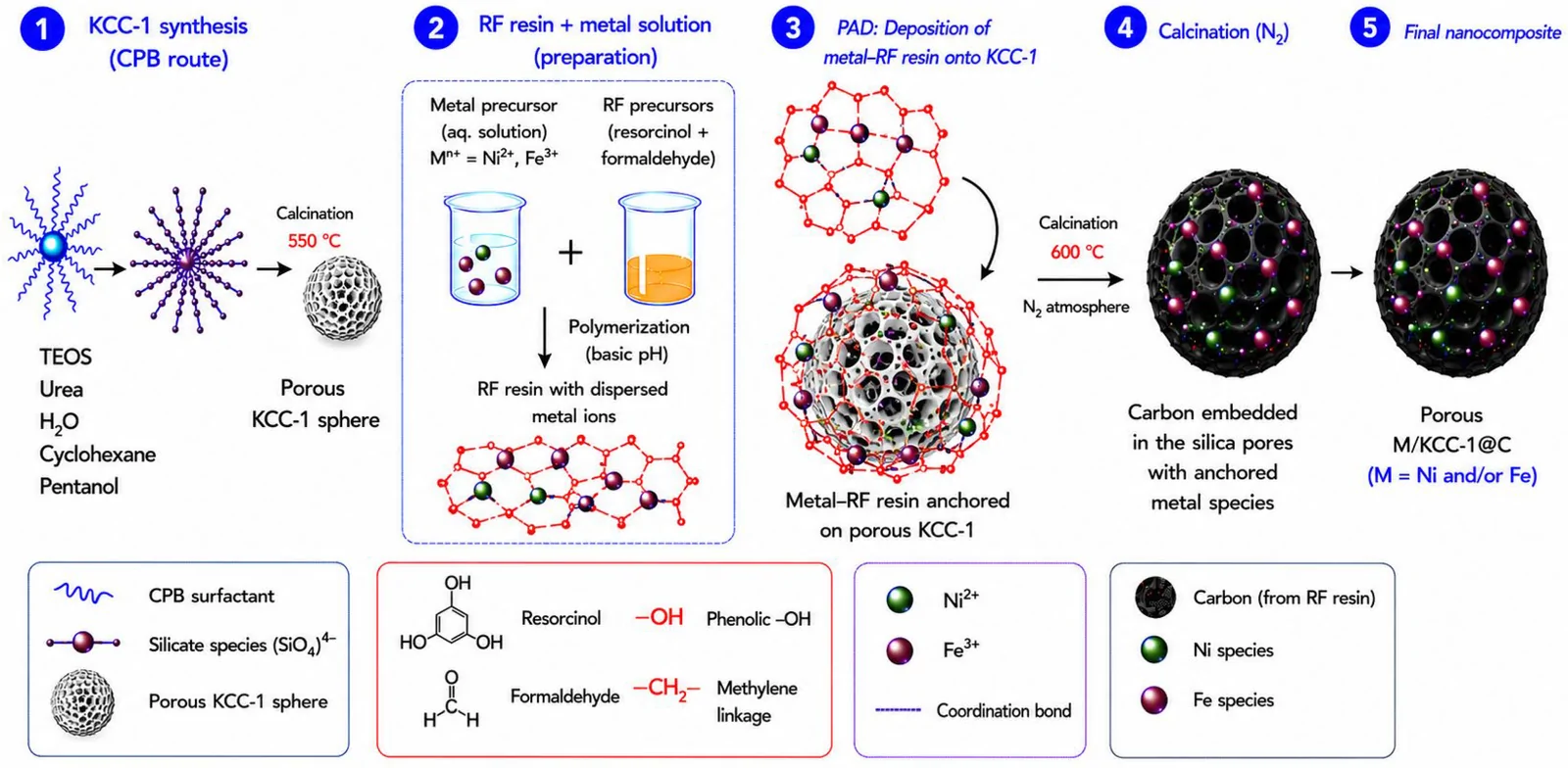
Schematic representation for the preparation of Fe, Ni and Fe-Ni supported in KCC-1@C nanocomposites.

Scheme 1 shows an illustration for the synthesis of KCC-1 support and 5 wt% (Ni, Fe, Ni-Fe)/KCC-1@C nanocomposites. Initially, CPB surfactant was used as a template to form a microemulsion. TEOS underwent a gradual hydrolysis process, resulting in the formation of negatively charged silicates in the presence of urea [27]. Urea was used in excess to increase the rate of hydrolysis of TEOS, promoting nucleation and thus seed formation in the initial stage of the reaction, as reported by Bayal et al. [19], which also contributes to the formation of smaller nanoparticles. Silicate condensation then took place, with silicates clustering in the space between the self-assembled template molecules [27]. Finally, it was calcined to remove the template molecules to obtain KCC-1 spheres. The distinctive feature of the PAD strategy employed in this work is the pre-complexation of the metal species with the preformed RF resin prior to its deposition onto KCC-1 [28]. Once the RF polymeric network is formed, its phenolic oxygen-containing functional groups can provide interaction sites for the metal cations introduced into the system, promoting their immobilization within the polymeric phase, generating a metal-containing RF precursor rather than a solution containing freely mobile metal ions [29,30].

This pre-complexation is particularly relevant to the deposition process because the RF resin acts as a molecular-scale carrier for the metal species. The interaction with the polymeric matrix spatially confines the metal precursors and reduces their mobility before and during deposition onto the KCC-1 surface [31]. Consequently, when the metal-containing RF phase is deposited onto the fibrous silica structure, the metal species are delivered together with the polymeric matrix and distributed throughout the support rather than being deposited independently as highly mobile ionic species. The RF layer therefore serves not only as a deposition medium but also as a confinement matrix that helps prevent localized accumulation of the metal precursors.

For the bimetallic Ni–Fe system, the same mechanism provides a common polymeric environment for both metal species, favoring their co-localization within the deposited RF phase. Upon subsequent thermal treatment, the RF matrix is pyrolyzed to form the carbonaceous layer, while the confined metal species undergo thermal transformation.

### 3.2. Structure and Chemical State of Carbon Matrix

**Figure 1:**
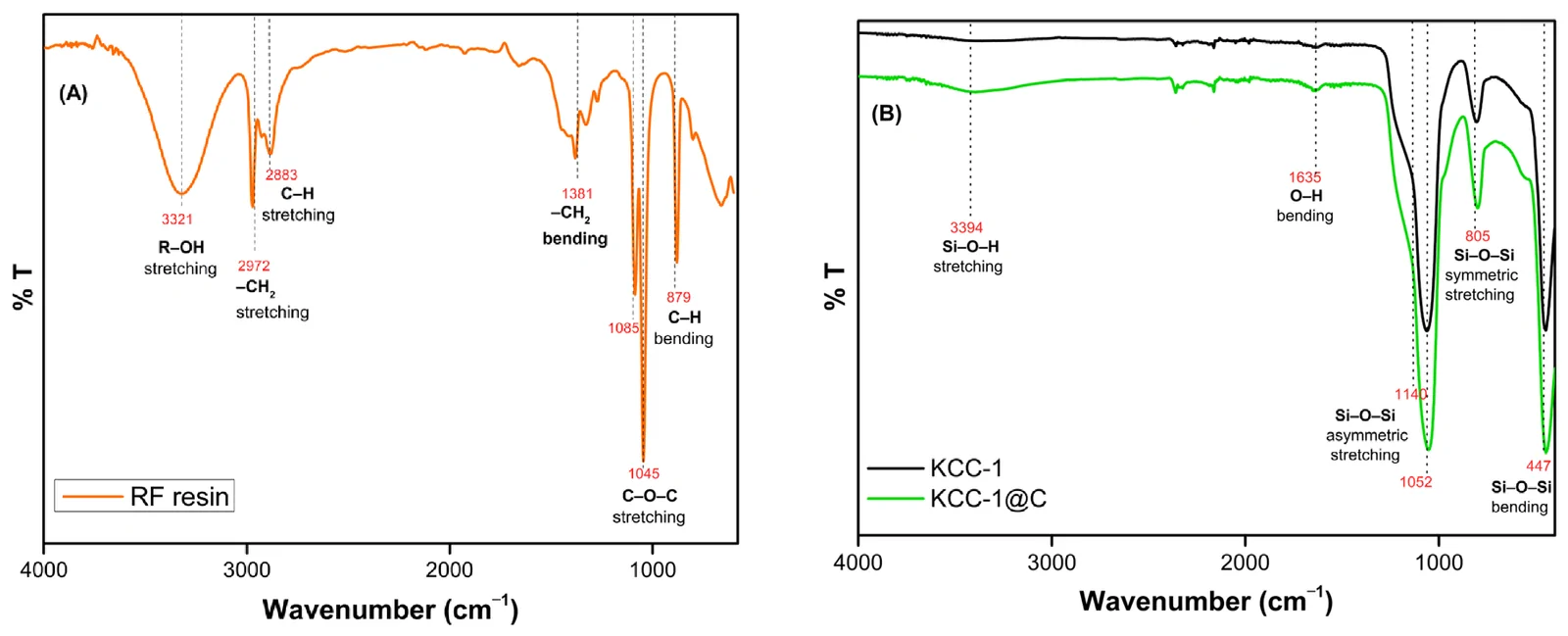
FTIR of (**A**) pure resin and (**B**) pure KCC-1 and RF-coated resin (KCC-1@C). The wavelength of the different bands is labeled in red.

The pure RF resin, pure dendritic fibrous nanosilica (KCC-1) and RF-coated resin (KCC-1@C) were studied by FTIR recorded from 4000 to 400 cm^−1^. In the spectra of RF polymer resin shown in Figure 1A, the broad peak around 3321 cm^−1^ is due to stretching vibration of hydroxyl groups, which is related to alcohol dilution and adsorbed water [32]. Peaks at 2972 and 2883 cm^−1^ are associated with the –CH_2_ and C–H stretching vibration of the methyl or methylene groups in the resin, which may be formed due to the condensation of resorcinol and formaldehyde. Moreover, the peak at 1381 cm^−1^ is typically associated with the H–C–H bending vibration [32]. Characteristic absorption peaks associated with C–O–C and C–O stretching peaks (1045 and 1085 cm^−1^) were observed. Finally, the peak at 879 cm^−1^ probably belongs to the C–H deformation vibration in formaldehyde [32]. Regarding the siliceous materials, both KCC-1 and KCC-1@C show a typical siliceous composition, as observed in Figure 1B. Characteristic peaks around 3394 cm^−1^ were assigned to surface-adsorbed water and all SiO–H types; vicinal, terminal and geminal silanols were identified [33]. The peak at 1635 cm^−1^ can be related to O–H bending, while the peaks ranging from 1052 to 1140 cm^−1^ could be assigned to the Si–O–Si asymmetric stretching vibration. Finally, the bands around 805 and 447 cm^−1^ can be associated with the Si–O–Si symmetric stretching vibration and the Si–O–Si bending mode, respectively.

**Figure 2:**
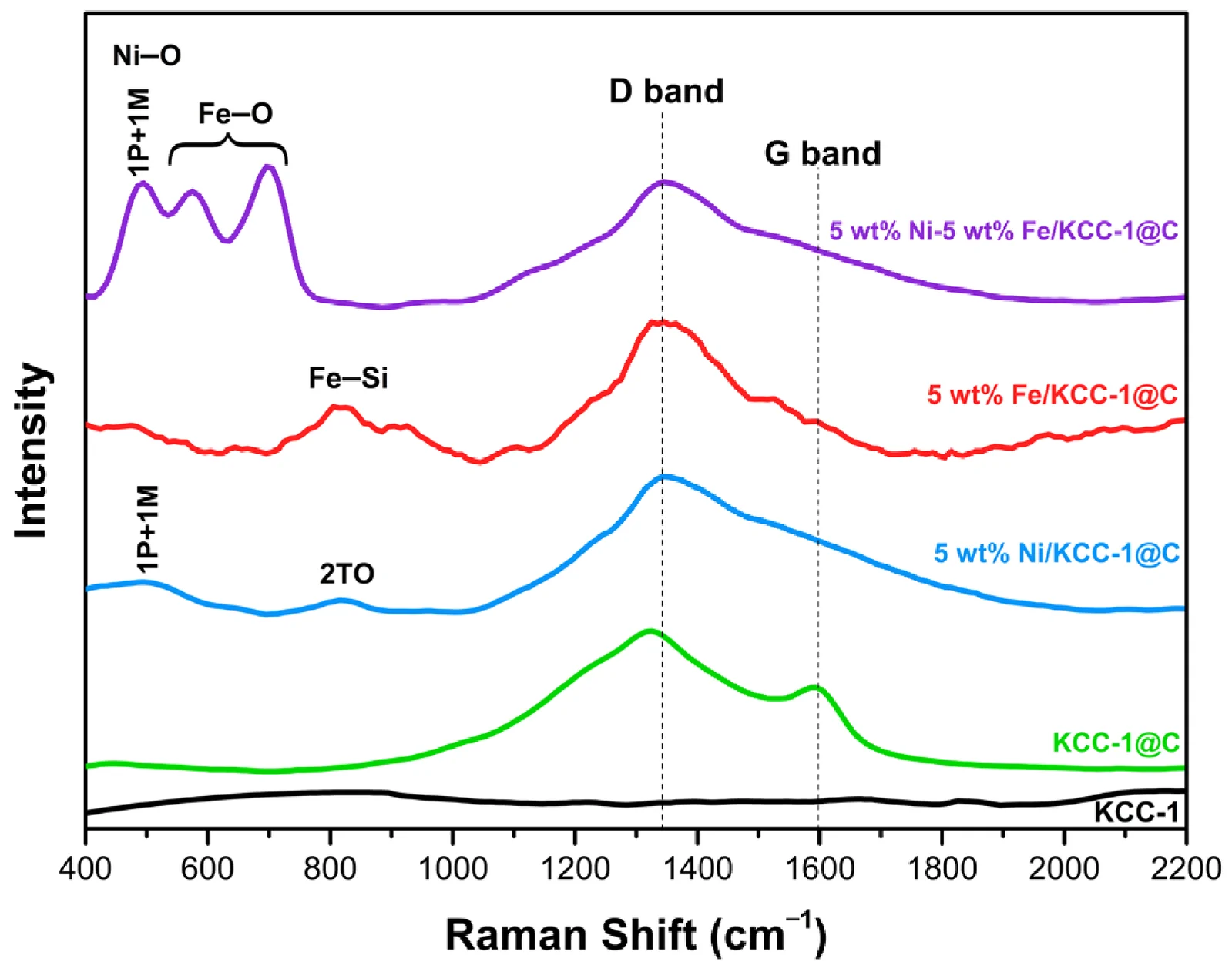
Raman spectra of KCC-1, KCC-1@C and nanocomposites (5 wt% Ni-5 wt% Fe/KCC-1@C, 5 wt% Fe-KCC-1@C, 5 wt% Ni-KCC-1@C).

Raman spectroscopy was used to confirm the dispersion of the carbon resin in the nanospheres. As shown in Figure 2, the carbonaceous support in KCC-1@C exhibited the Raman bands at 1324 and 1594 cm^−1^, which are attributed to the D and G bands of graphite, respectively. The G band corresponds to the vibration of sp^2^-bonded carbon in crystalline graphite, while the D band is associated with the crystal disorder in carbon. The intensity ratio of D-band to G-band (ID/IG) is indicative of the degree of disorder of the graphitic lattice [34]. In the case of KCC-1@C, the ID/IG is 1.7, which is indicative of a high degree of disorder [35,36].

The Raman spectra of the mono- and bimetallic nanocomposites exhibit a prominent and broad D band around 1350 cm^−1^, with the characteristic G band either absent or significantly attenuated. This spectral behavior suggests a disordered carbon structure, likely induced by the incorporation of metal species, which disrupt the organization of the carbonaceous matrix [25]. Additionally, the D band in the nanocomposites shows a slight shift relative to the KCC-1@C reference, along with reduced intensity and peak broadening, features commonly associated with amorphous hydrogenated carbon [37]. This is consistent with the nature of carbon derived from resorcinol–formaldehyde resin, which typically contains a high fraction of sp^3^-hybridized carbon due to residual hydrogen, leading to a dominant D band and suppression of G band features [35].

In addition, Raman spectroscopy also confirmed the presence of iron and nickel in the metal-doped nanospheres; the mono- and bimetallic composites exhibit a series of bands between 400 and 900 cm^−1^. The Raman spectrum for the bimetallic 5 wt% Ni-5 wt% Fe/KCC-1@C nanocomposite shows a band around 494 cm^−1^, which can be attributed to the nanometric nickel oxide formed on the carbonaceous surface (1 phonon + 1 magnon scattering, 1P + 1M). Although NiO has a cubic structure and is Raman inactive, due to defects or particle size at the nanoscale or structural disorder, single phonon or single magnon, Raman modes can become active [38]. The bimetallic composite also exhibits two bands around 574 cm^−1^ and 694 cm^−1^ that have been tentatively assigned to wüstite (FeO). Although wüstite presents a very weak Raman scattering, two modes have been reported in the literature; one around 595 cm^−1^ related to inelastic second harmonic light scattering process [39] and another at 652 cm^−1^, a band similar to those observed in magnetite [40]. The variation between the two bands obtained experimentally and those reported in the literature can be related to different reasons. Wüstite is a non-stoichiometric iron oxide with an approximate composition between Fe_0.84_O and Fe_0.95_O [40]. In addition, it is metastable and can present disproportionation to metallic iron and magnetite, presenting changes in the wavenumbers of the Raman spectrum [41].

The monometallic composites present very weak signals in the range of 400 cm^−1^ and 900 cm^−1^; this can be justified by the lower amount of metal compared to the bimetallic ones. For the nickel composite 5 wt% Ni/KCC-1@C, two weak bands were observed at 494 cm^−1^ and 814 cm^−1^; the first one was associated with the 1P + 1M scattering discussed previously and the second band was assigned to the transverse mode (2TO) of nickel oxide [42]. The Raman band observed at 820 cm^−1^ in the 5 wt% Fe/KCC-1@C composite suggests the formation of an Fe–Si interaction, likely Fe_2_SiO_4_ [43].

### 3.3. Phase Composition and Valence State of Metal Species

**Figure 3:**
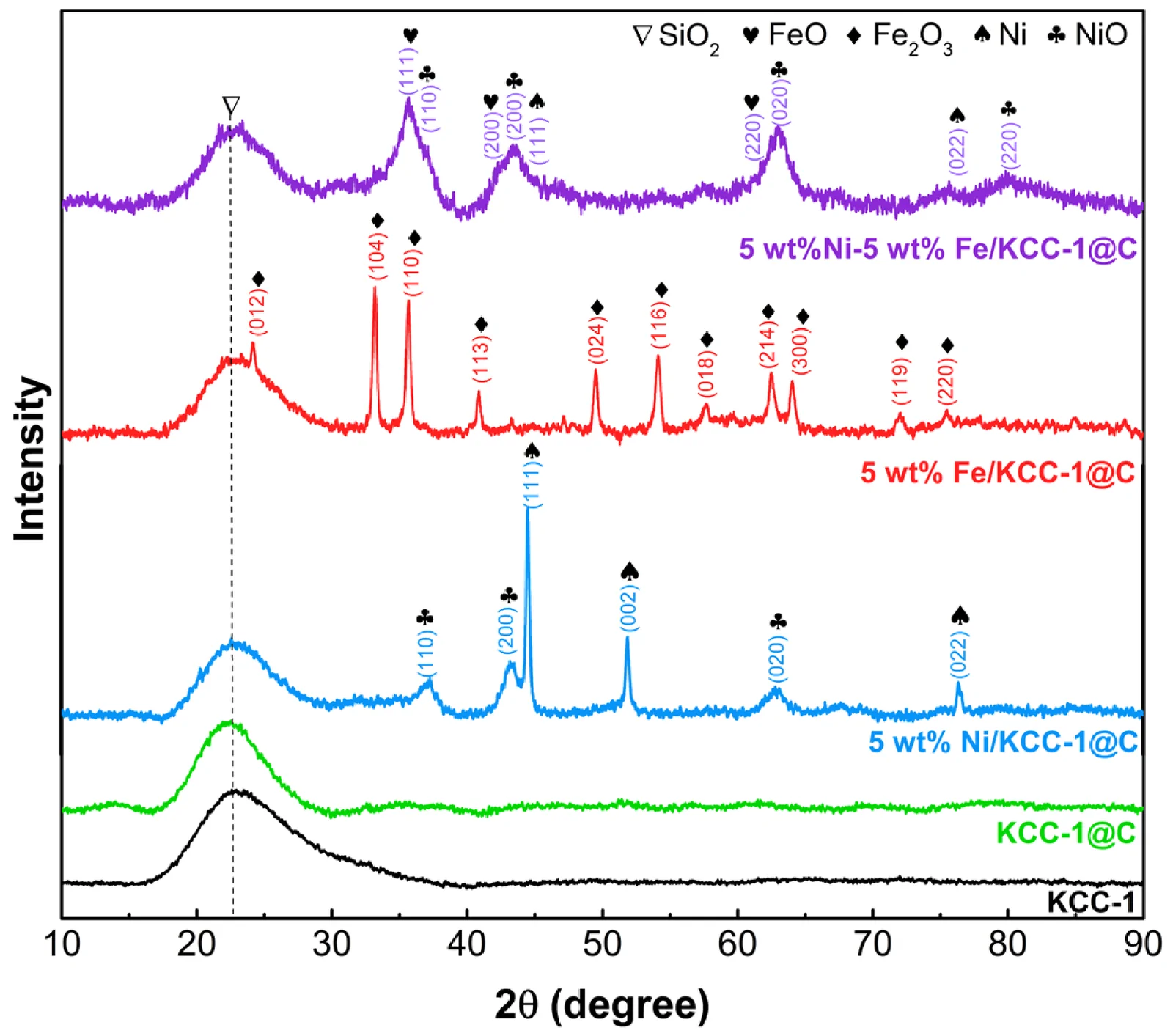
XRD patterns of KCC-1, KCC-1@C and nanocomposites (5 wt% Ni-5 wt% Fe/KCC-1@C, 5 wt% Fe-KCC-1@C, 5 wt% Ni-KCC-1@C).

The XRD patterns of the metal-functionalized samples supported on KCC-1@C show significant variations depending on the metal composition (Figure 3). All samples exhibit a broad peak between 20° and 30° (2θ), corresponding to amorphous silica, consistent with reported patterns for silica nanospheres [44]. In the case of the monometallic iron sample (5 wt% Fe/KCC-1@C), diffraction peaks characteristic of hematite (Fe_2_O_3_) are observed, as shown in Figure 3, indicating that iron is predominantly stabilized in the +3 oxidation state under the applied thermal treatment. In the monometallic nickel sample (5 wt% Ni/KCC-1@C), diffraction peaks appear at 2θ = 36.95°, 43.33°, and 62.82°, corresponding to the (110), (200), and (020) planes of nickel oxide (NiO), along with peaks at 2θ = 44.50°, 51.86°, and 76.34°, assigned to the (111), (002), and (022) planes of cubic metallic nickel (Ni^0^). This indicates partial reduction of NiO, probably promoted by reducing gases generated during the carbonization of the resorcinol–formaldehyde resin at 600 °C under a nitrogen atmosphere [45].

In the bimetallic system (5 wt% Ni–5 wt% Fe/KCC-1@C), broader diffraction peaks are observed. The maxima of several of these peaks coincide with the expected positions of NiO reflections. However, shoulders or additional contributions within the broad peaks are also consistent with wüstite (FeO) and metallic Ni. Because of the extensive peak overlap, these assignments are further evaluated using the complementary characterization results presented below. Overall, this behavior suggests that nickel facilitates the partial reduction of iron while itself remaining in the oxidized state [45]. The notable broadening of the diffraction peaks in this sample may indicate smaller crystallite sizes and/or higher dispersion of metallic phases on the support, likely due to strong metal–metal interactions during the thermal synthesis and the formation of highly amorphous or poorly crystalline phases [46,47].

Since iron oxides exhibit very small differences in lattice parameters and given the small particle size and amorphous nature of the samples, X-ray diffraction (XRD) alone is often insufficient. Therefore, it is essential to complement XRD with an iron oxidation state-sensitive technique.

**Figure 4:**
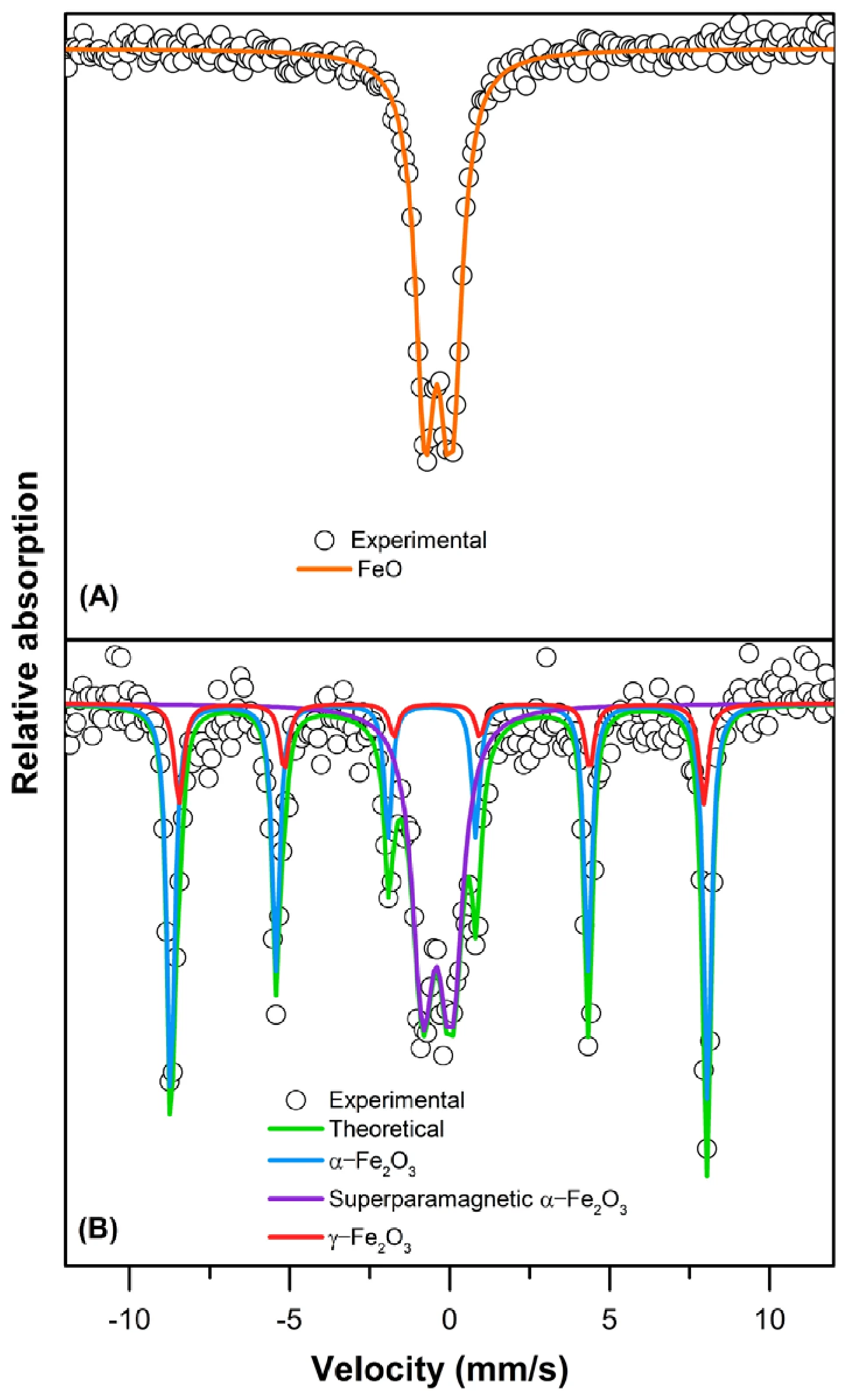
Mössbauer spectra of iron-containing materials (**A**) 5 wt% Ni-5 wt% Fe/KCC-1@C and (**B**) 5 wt% Fe/KCC-1@C.

To complement the XRD structural analysis and unequivocally identify the chemical environment and oxidation states of the iron species, room-temperature Mössbauer spectroscopy was performed. Figure 4 shows the room temperature Mössbauer spectra of (A) 5 wt% Ni–5 wt% Fe/KCC-1@C and (B) 5 wt% Fe/KCC-1@C. The Mössbauer parameters derived from these spectra are summarized in Table 1. The spectrum of the bimetallic sample (5 wt% Ni–5 wt% Fe/KCC-1@C) exhibits a central doublet with a low isomer shift (IS) of approximately 0.33 mm/s, characteristic of Fe^2+^ in a paramagnetic environment, which is assigned to the wüstite phase [48,49]. This result is consistent with the XRD analysis, where broad peaks corresponding to FeO were also identified. The exclusive presence of FeO in the Mössbauer spectrum confirms the partial reduction of Fe^3+^ from initial Fe_2_O_3_ to Fe^2+^ (FeO), ruling out the formation of mixed spinel-type phases such as NiFe_2_O_4_, where iron would remain as Fe^3+^. This phase evolution is consistent with a partial reduction of Fe_2_O_3_ obtained from the Fe(NO_3_)_3_·9H_2_O precursor during the carbonization of the resorcinol–formaldehyde resin in a nitrogen atmosphere. The pyrolysis process releases reducing gases (e.g., CO, H_2_), creating localized reducing environments that, in the presence of a hydrogenation catalyst such as nickel, can promote the efficient transformation of Fe^3+^ to Fe^2+^ species (FeO) [50,51,52]. Furthermore, this stabilized FeO phase is intimately linked to the encapsulation of the iron species within the RF-derived carbon layer. The surrounding carbon matrix acts as a physical barrier that restricts the exposure of Fe^2+^ species to ambient oxygen, thereby preventing re-oxidation back to Fe^3+^ oxides. Concurrently, this “carbon-coated metal” architecture induces a pronounced spatial confinement effect. By physically entrapping the iron nanoparticles within the carbonaceous shell and the dendritic mesopores of KCC-1, particle migration and thermal sintering are effectively suppressed during high-temperature carbonization, preserving well-dispersed, nanoscale FeO domains.

In contrast, the Mössbauer spectrum of the monometallic iron catalyst (5 wt% Fe/KCC-1@C) displays two sextets and one central doublet, indicating the coexistence of multiple iron oxide phases. The first sextet, with parameters δ = 0.411 mm/s, ΔE_Q_ = 0.226 mm/s, and B_hf_ = 518 kOe, corresponds to hematite (α-Fe_2_O_3_) [53], while the second sextet, with δ = 0.298 mm/s, ΔE_Q_ = 0.140 mm/s, and B_hf_ = 507 kOe, is attributed to maghemite (γ-Fe_2_O_3_) [54,55,56]. Additionally, a central doublet with δ = 0.343 mm/s, ΔE_Q_ = 0.819 mm/s, and B_hf_ = 0 is assigned to the superparamagnetic (SPM) α-Fe_2_O_3_ phase [57]. The presence of maghemite suggests the prior formation of magnetite (Fe_3_O_4_) as an intermediate phase, which was subsequently oxidized under the synthesis conditions or upon exposure to air. Unlike the 5 wt% Ni–5 wt% Fe/KCC-1@C nanocomposite, the absence of Ni can affect the efficient reduction process promoted by the reductive conditions mentioned above, thus facilitating reoxidation from FeO and Fe_3_O_4_ (incomplete reduction species) to Fe^3+^ oxides depending on local oxygen availability. The coexistence of crystalline and superparamagnetic iron oxide phases [58,59], confirms the presence of nanoscale particles and complex redox dynamics during synthesis.

### 3.4. Morphology and Elemental Distribution

**Figure 5:**
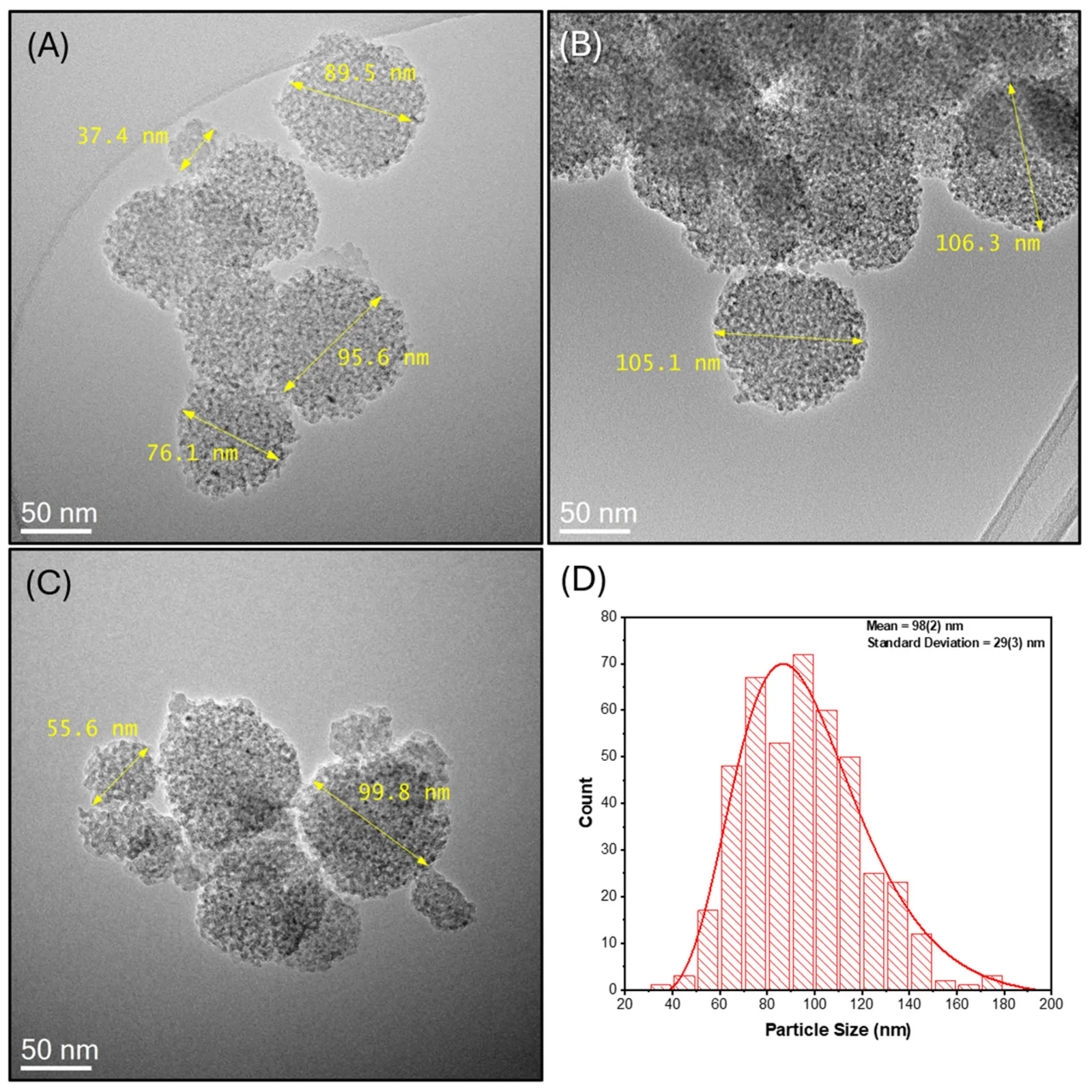
Intermediate-magnification TEM image of a (**A**) 5 wt% Fe/KCC-1@C, (**B**) 5 wt% Ni/KCC-1@C and (**C**) 5 wt% Fe/KCC-1@C specimen. (**D**) Size distribution of the KCC-1 supports, fitted with a log-normal distribution function, extracted from TEM images.

**Figure 6:**
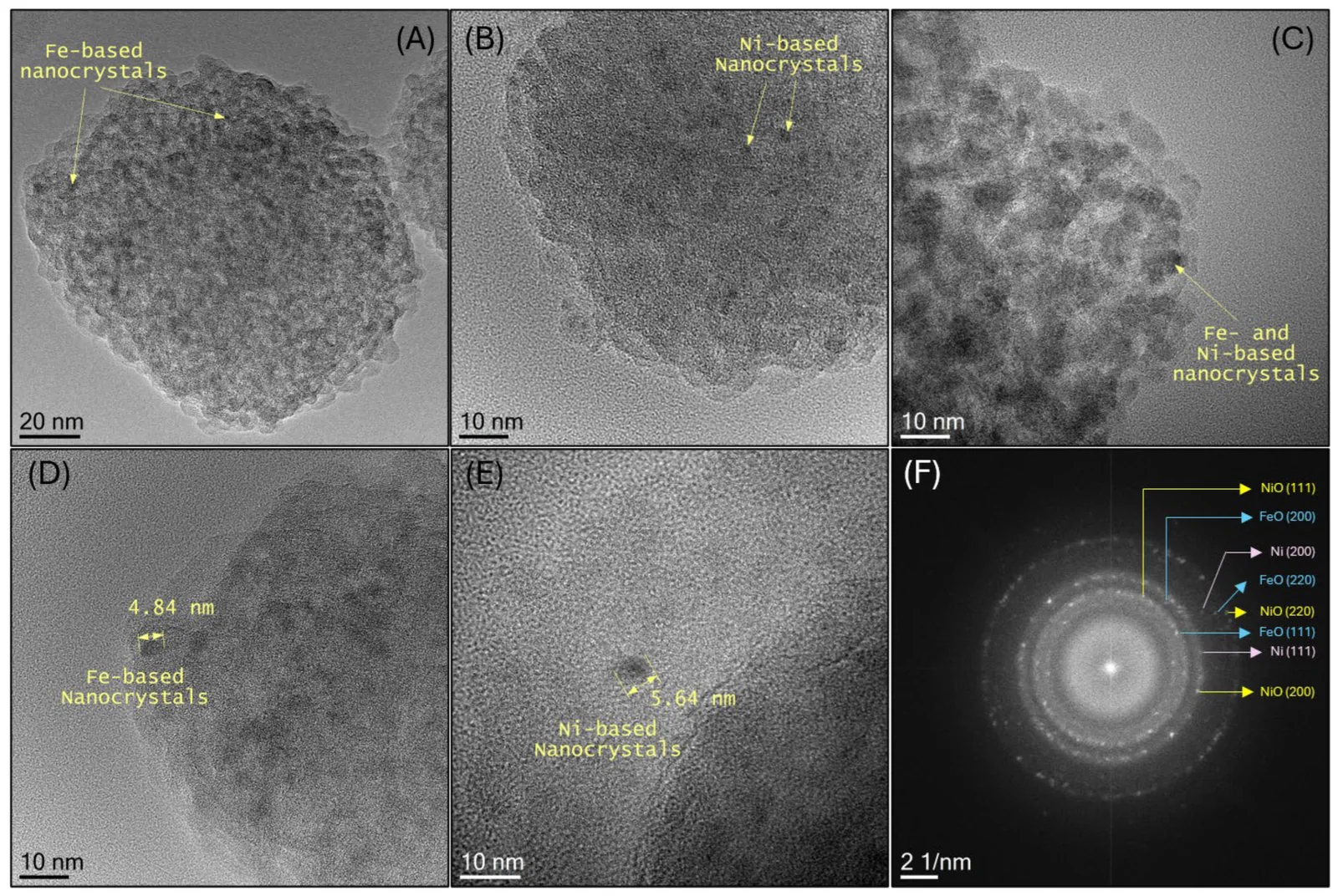
HRTEM images acquired in the mono- and bimetallic nanocomposites: (**A**,**D**) for the 5 wt% Fe/KCC-1@C, (**B**,**E**) for the 5 wt% Ni/KCC-1@C, (**C**) for the 5 wt% Ni-5 wt% Fe/KCC-1@C. (**F**) FFT image extracted from (**C**).

The dispersion and size distribution of the KCC-1-based supports in all nanocomposites were analyzed using a series of low- and high-magnification TEM images recorded for all samples. Representative TEM images of the three specimens are shown in Figure 5, revealing that the supports are fibrous dendritic silica nanostructures, with quasi-spherical morphology, and diameters ranging from 50 to 140 nm, as illustrated in the accompanying histogram. The average support size was estimated by fitting the size distribution histogram with a log-normal distribution function, yielding a mean size of 98 nm and a standard deviation of 29 nm. HRTEM images shown in Figure 6 illustrate the amorphous surface morphology typical of silica-based nanostructures [60]. Darker contrast regions on the nanostructures correspond to the impregnated metal-containing phases (Figure 6A–C), which appear well-dispersed on the carbon-coated KCC-1 fibrous surface. The observed rough surface texture is attributed to the formation of disordered amorphous carbon, which allowed the dispersion of the metal oxides. The size of the nanoparticles was estimated from HRTEM images by analyzing isolated nanocrystals located near the edges of the support (Figure 6D,E). Both mono- and bimetallic nanocomposites were found to contain Fe- and Ni-based nanocrystals with sizes ranging from 3 to 6 nm. The nanocrystalline nature of the deposited metal species was further confirmed by digital electron diffraction patterns obtained via fast Fourier transform (FFT) of HRTEM images (Figure 6F). The presence of concentric diffraction rings with multiple discrete spots indicates the existence of randomly oriented nanocrystals [61]. The reciprocal-space radii measured for representative diffraction spots are consistent with reflections expected for NiO, metallic Ni, and FeO.

**Figure 7:**
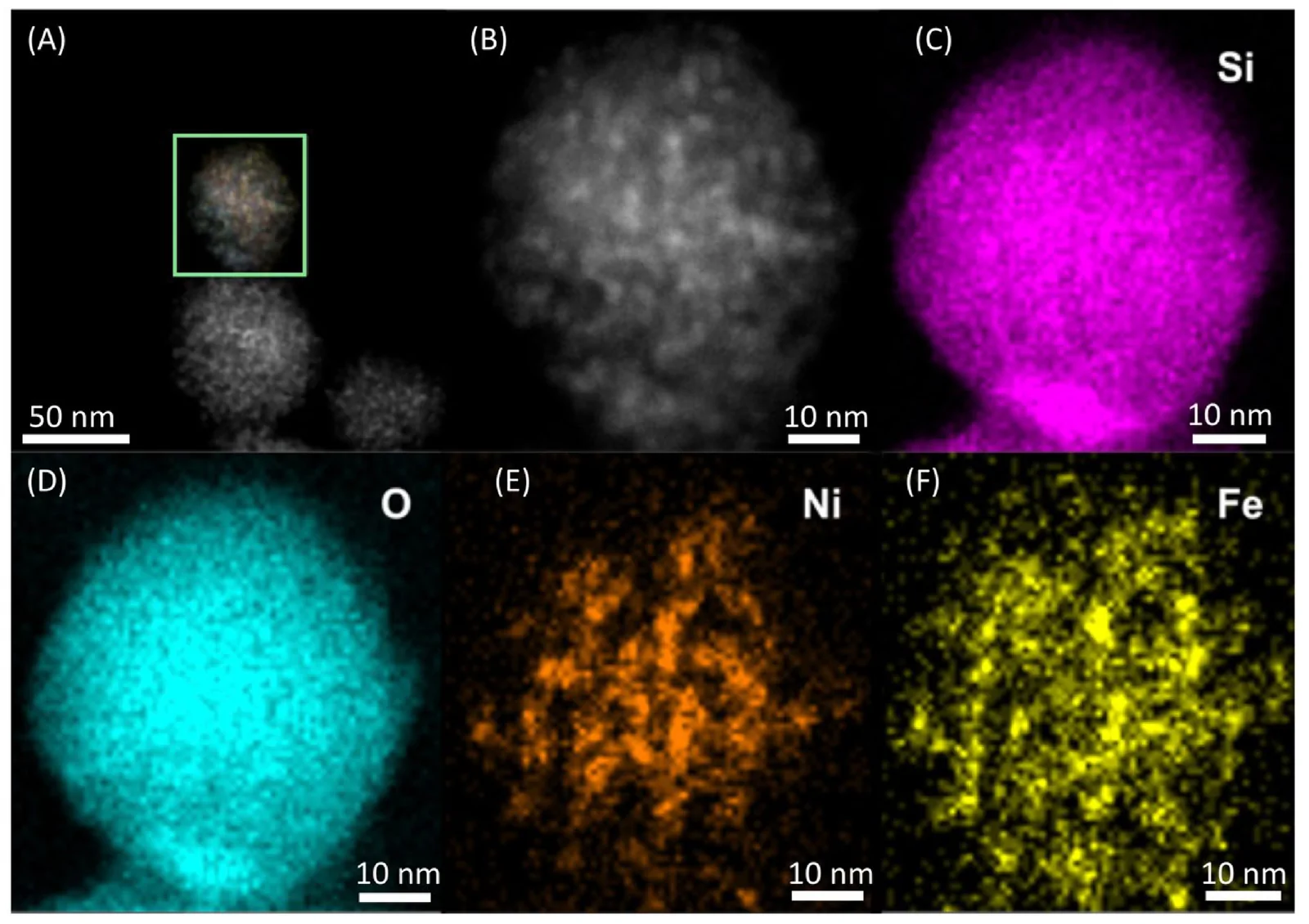
(**A**) HAADF-STEM image of the 5 wt% Ni-5 wt% Fe/KCC-1@C nanocomposite. (**B**–**F**) STEM-EDS chemical maps of the particle marked in (**A**) with a green square, which includes the simultaneously acquired (**B**) HAADF signal, and the elemental maps for (**C**) Si, (**D**) O, (**E**) Ni and (**F**) Fe.

To evaluate the spatial distribution of Fe and Ni species in the bimetallic nanocomposite, STEM-EDS chemical mapping was conducted on a 5 wt% Ni-5 wt% Fe/KCC-1@C specimen (Figure 7A,B). Elemental maps for Si and O (Figure 7C,D) show a homogeneous distribution, consistent with the SiO_2_ support. In contrast, elemental maps for Fe and Ni (Figure 7E,F) reveal weaker and non-uniform signals, suggesting the localized presence of the metallic species in the form of discrete nanoparticles. A non-complete overlapping between the Fe and Ni maps confirms that the impregnated species are spatially distinct, supporting the bimetallic nature of the composite without forming an Fe–Ni alloy. These morphological observations are in good agreement with the structural findings from XRD and Mössbauer spectroscopy, which indicated the presence of separate Fe- and Ni-based phases without alloy formation.

### 3.5. Textural Properties and Thermal Behavior

**Figure 8:**
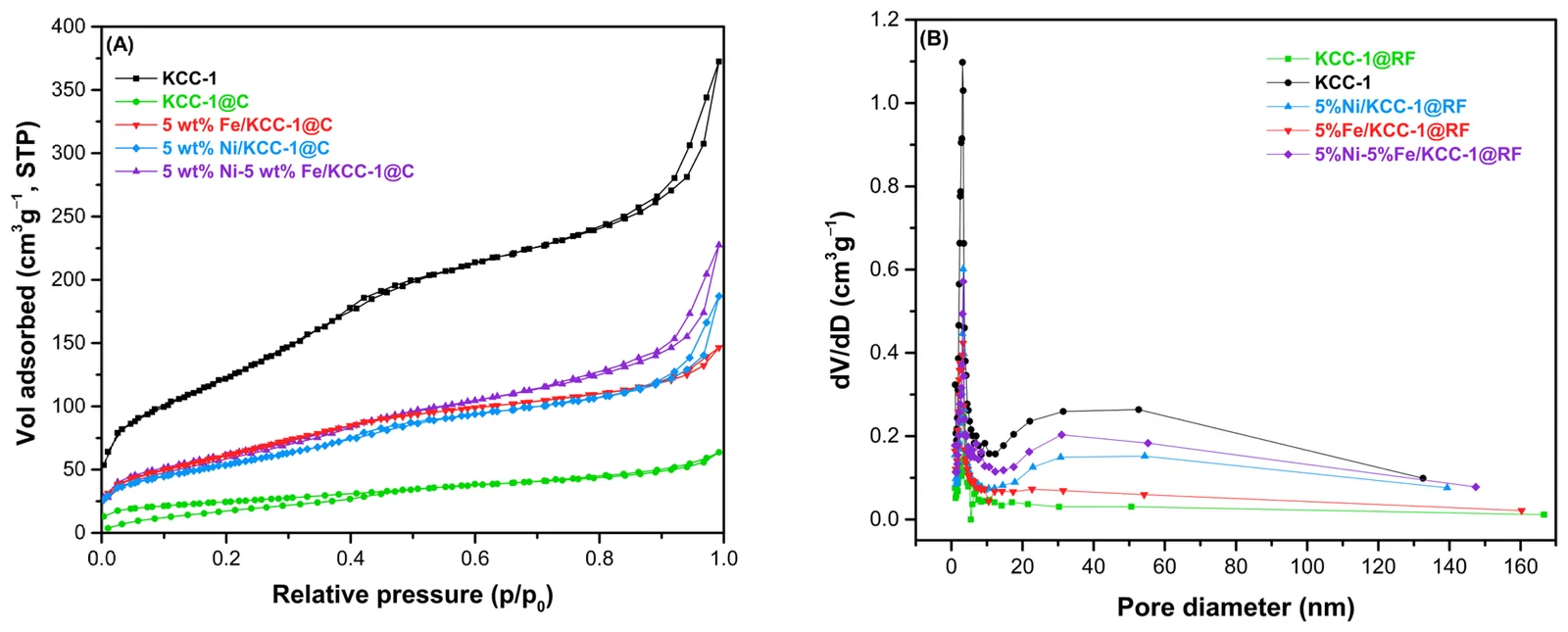
(**A**) N_2_ adsorption–desorption isotherm and (**B**) pore diameter of KCC-1, KCC-1@C and Ni- and Fe-based nanocomposites.

The textural properties of the synthesized materials were evaluated by N_2_ adsorption–desorption isotherms at 77 K, as shown in Figure 8, and the corresponding BET surface area, pore volume, and average pore diameter are summarized in Table 2. Pristine KCC-1 exhibits a Type IV isotherm with a pronounced H1-type hysteresis loop (IUPAC classification), indicative of mesoporous materials with uniform, cylindrical pores and open fibrous morphology [62]. Its high BET surface area (462.7 m^2^·g^−1^), large pore volume (0.629 cm^3^·g^−1^) and average pore diameter (3.12 nm) are consistent with its known hierarchical mesostructure. After coating, the (KCC-1@C) isotherm changes drastically to a Type II profile, showing a marked reduction in nitrogen uptake and virtually no hysteresis. This behavior is attributed to pore blockage by the polymeric carbon layer, which significantly decreases surface area (84.8 m^2^·g^−1^), pore volume (0.127 cm^3^·g^−1^) and pore diameter (1.01 nm), indicating a partial collapse or obstruction of mesopores.

In the metal-functionalized systems, where Fe^3+^ and/or Ni^2+^ were pre-mixed with the RF resin prior to deposition on KCC-1, and subsequently carbonized, a recovery of mesoporosity is observed. All metal-containing samples display Type IV isotherms with H3-type hysteresis loops, typical of materials with slit-shaped pores and non-uniform mesostructures [63]. This suggests that the incorporation of metal precursors into the resin matrix prior to deposition on the support leads to the development of a distinct porous structure during carbonization, likely associated with phase separation, gas evolution, and structural rearrangements that generate mesoporous domains [64].

Quantitatively, the monometallic Fe-based sample (5 wt% Fe/KCC-1@C) shows a BET area of 197.6 m^2^·g^−1^, indicating partial recovery of porosity compared to KCC-1@C, likely due to Fe-related decomposition or void formation within the carbon matrix. The Ni-based sample (5 wt% Ni/KCC-1@C) exhibits a slightly higher surface area (223.6 m^2^·g^−1^) and pore volume (0.213 cm^3^·g^−1^) compared to the Fe-containing system, suggesting that nickel may facilitate more homogeneous carbon structure formation. Remarkably, the bimetallic sample (5 wt% Ni-5 wt% Fe/KCC-1@C) presents the highest surface area among the functionalized materials (276.4 m^2^·g^−1^) and a pore volume of 0.316 cm^3^·g^−1^, indicating a synergistic effect between Ni and Fe that enhances pore development or stabilization during the thermal treatment [65].

These findings clearly show that the textural properties of the final hybrid materials are strongly influenced by the synthetic strategy, particularly the co-impregnation of metal precursors into the resin prior to its deposition on the KCC-1 support. The bimetallic approach not only promotes better dispersion of the active phases but also contributes to the preservation and regeneration of the mesoporous network [66,67].

**Figure 9:**
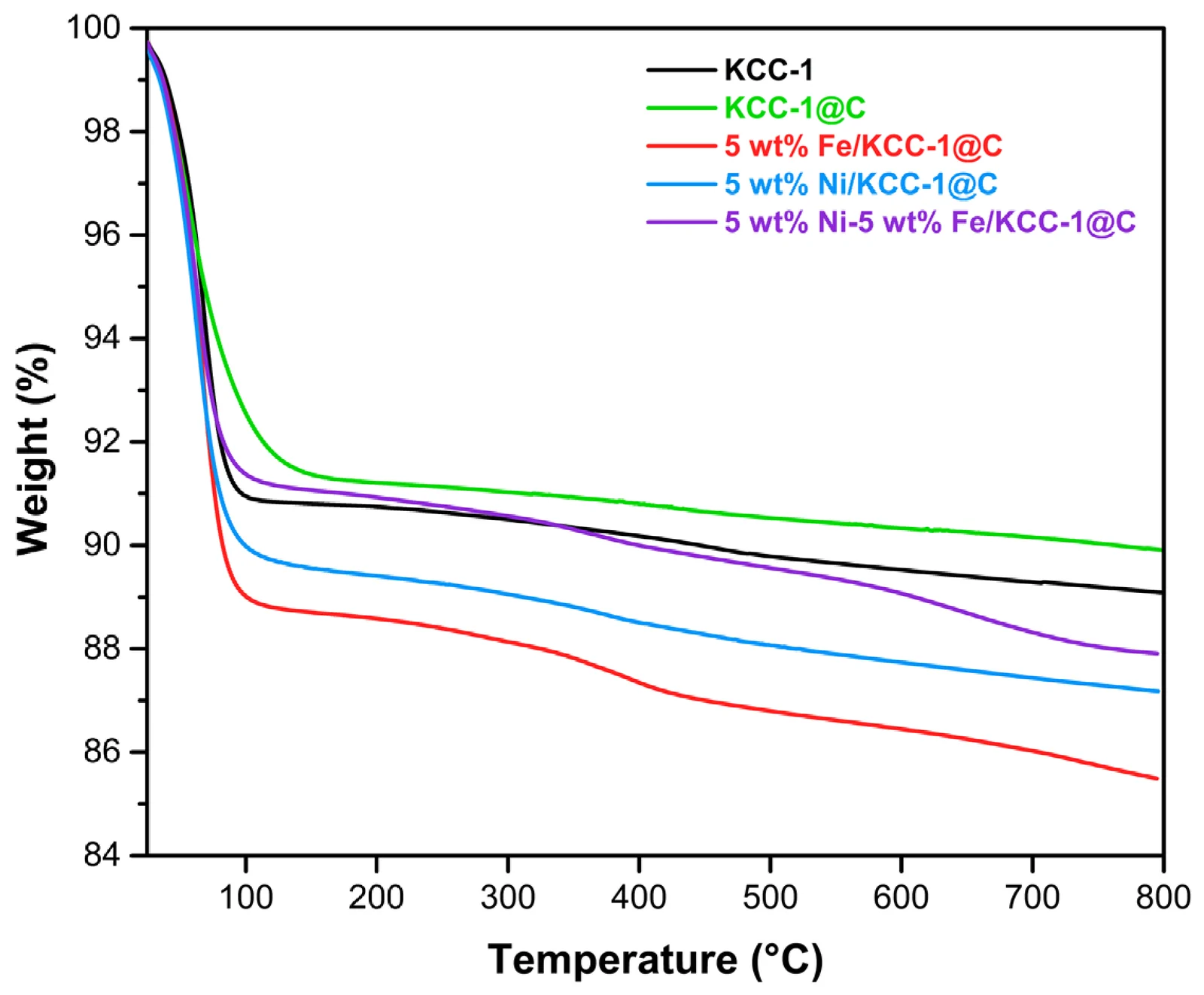
TGA curve of KCC-1, KCC-1@C and nanocomposites (5 wt% Fe-KCC-1@C, 5 wt% Ni-KCC-1@C, 5 wt% Ni-5 wt% Fe/KCC-1@C).

The thermal behavior and structural stability of the synthesized materials were evaluated by TGA under a nitrogen atmosphere from room temperature to 800 °C at a heating rate of 5 °C·min^−1^. As shown in Figure 9, all profiles exhibit a two-step weight loss process, with total weight losses ranging between 10% and 14.5% across the entire temperature range. The first and primary weight loss event occurs below 100 °C, accounting for approximately 8–11% of the total mass reduction in all samples. This initial drop is predominantly attributed to the desorption of physisorbed water and atmospheric moisture retained within the mesoporous structure of KCC-1, which is favored by the presence of hydrophilic surface silanol groups [16]. Beyond 100 °C, the weight loss profile shows a small but progressive decrease for all samples, with a minor secondary mass loss between 100 °C and 800 °C (~2–4%). This minimal mass reduction is mainly associated with the condensation of remaining silanol species and the evolution of residual oxygenated surface groups from the carbon matrix. Interestingly, the total weight loss above 100 °C shows a slight decrease for the monometallic Ni-based nanocomposite (~2.8%) compared to the monometallic Fe-based counterpart (~4.0%) and the bimetallic system (~3.8%) reflects the distinct thermal evolution of the individual metallic species, where the presence of nickel—both in the monometallic and bimetallic systems—appears to modulate the surface group decomposition compared to the Fe-only counterpart [8,68].

## 4. Conclusions

Hybrid nanocomposites based on dendritic fibrous silica (KCC-1) and RF resin were successfully synthesized using the PAD method for the incorporation of Ni and Fe, in mono- and bimetallic configurations. The functional groups in the RF resin facilitated uniform distribution of Fe- and Ni-based nanoparticles, while the porous architecture of KCC-1 served as a favorable support. FTIR and Raman spectroscopy confirmed the presence of amorphous carbon and metal–oxygen interactions, along with structural modifications in the carbon matrix induced by metal incorporation. XRD analysis revealed hematite (α-Fe_2_O_3_) in the Fe-based system, a combination of NiO and Ni^0^ in the Ni-based system, and a mixture of FeO, NiO and Ni in the bimetallic material. These findings were supported by Mössbauer spectroscopy, which detected Fe^3+^ in the monometallic and Fe^2+^ in the bimetallic system. HRTEM and EDS mapping confirmed homogeneous dispersion of metal-based nanoparticles (between 3–6 nm) within the carbon-coated silica spheres. Although metal and resin loading reduced the BET surface area, the nanocomposites maintained mesoporosity with Type IV isotherms and H3-type hysteresis, displaying thermal stability up to 800 °C as observed for other DFNS previously reported.

## Figures and Tables

**Table 1:** The parameters derived from the Mössbauer spectra of 5 wt% Ni-5 wt% Fe/KCC-1@C and 5 wt% Fe/KCC-1@C.

Sample	Phase	*δ*/mm s^−1^	ΔE_Q_/mm s^−1^	B_hf_/kOe	W/mm s^−1^	RI/%
5 wt% Ni-5 wt% Fe/KCC-1@C	FeO	−0.33	0.742	0	0.365	100
5 wt% Fe/KCC-1@C	α-Fe_2_O_3_	−0.411	0.226	518	0.145	44.9
	α-Fe_2_O_3_ (SPM)	−0.343	0.819	0	0.393	41.8
	γ-Fe_2_O_3_	−0.298	0.140	507	0.170	13.3

Isomer shift (*δ*), quadrupole splitting (ΔE_Q_), hyperfine field (B_hf_), width (W) in mm s^−1^, and relative intensities (RI) in percent.

**Table 2:** Texture Properties of Ni-Fe mono- and bimetallic NPs supported on KCC-1@C.

Samples	^a^ BET Surface Area (m^2^·g^−1^)	^b^ Pore Volume (cm^3^·g^−1^)	^b^ Pore Diameter (nm)
KCC-1	462.689	0.629	3.12
KCC-1@C	84.771	0.127	1.01
5 wt% Ni/KCC-1@C	197.644	0.309	3.30
5 wt% Fe/KCC-1@C	232.183	0.253	1.05
5 wt% Ni-5 wt% Fe/KCC-1@C	226.040	0.386	3.30

^a^ Calculated by the BET method. ^b^ Determined by BJH method.

## Data Availability

The original contributions presented in this study are included in the article. Further inquiries can be directed to the corresponding authors.

## Abbreviations

The following abbreviations are used in this manuscript:

BET	Brunauer–Emmett–Teller
BJH	Barrett–Joyner–Halenda
CENM	Centro de Excelencia en Nuevos Materiales
CPB	Cetylpyridinium Bromide
CTAB	Cetyltrimethylammonium Bromide
DFNS	Dendritic Fibrous Nanosilica
EDS	Energy-Dispersive X-ray Spectroscopy
FFT	Fast Fourier Transform
FT-IR	Fourier Transform Infrared Spectroscopy
GC	Gas Chromatography
GIFA	Grupo de Investigación en Física Aplicada
GMTF	Grupo de Metalúrgica Física y Teoría de Transiciones de Fase
GPD	Grupo de Películas Delgadas
HAADF	High-Angle Annular Dark Field
HRTEM	High-Resolution Transmission Electron Microscopy
ICTS	Infraestructuras Científicas y Técnicas Singulares
ID/IG	D-band to G-band intensity ratio
INMA	Instituto de Nanociencia y Materiales de Aragón
IS	Isomer Shift
IUPAC	International Union of Pure and Applied Chemistry
KCC-1	Fibrous dendritic silica nanospheres
LMA	Laboratorio de Microscopias Avanzadas
MCM-41	Mobil Composition of Matter No. 41
MOSFIT	Mössbauer Spectrum Fitting Software
MS	Mass Spectrometer
MYSNs	Mesoporous Yolk–Shell Nanoparticles
PAA	Polyacrylic Acid
PAD	Polymer-Assisted Deposition
PEI	Polyethyleneimine
RF	Resorcinol–Formaldehyde
RI	Relative Intensity
SBA-15	Santa Barbara Amorphous-15
STEM	Scanning Transmission Electron Microscopy
SPM	Superparamagnetic
TEM	Transmission Electron Microscopy
TEOS	Tetraethyl Orthosilicate
TGA	Thermogravimetric Analysis
XRD	X-ray Diffraction
